# *Corynebacterium bovis* infection after autologous fat grafting in breast augmentation: a case report

**DOI:** 10.3389/fcimb.2023.1265872

**Published:** 2023-12-08

**Authors:** Xin You, Yao Yao, JianHua Gao, YunJun Liao

**Affiliations:** Department of Plastic and Cosmetic Surgery, Nanfang Hospital, Southern Medical University, Guang Zhou, Guang Dong, China

**Keywords:** fat graft, breast augmentation, *Corynebacterium bovis*, high-throughput DNA sequencing, bacterial infection

## Abstract

In this report, we present a case study of a rare human bacterium, *Corynebacterium bovis*, which caused an infection in a patient who had undergone autologous fat-based breast augmentation using cryopreserved fat. This infection occurred during a secondary fat grafting procedure. To identify the bacteria causing the infection, we used high-throughput DNA sequencing technology since this bacterium is seldomly reported in human infections. The patient was successfully treated with intravenous imipenem. We also discuss potential factors that may have contributed to this unusual bacterial infection and propose that DNA sequencing can be a useful tool in cases where standard culture techniques fail to identify the causative agent. Additionally, we highlight the importance of further research on the cryopreservation of fat. In summary, this case highlights the possibility of rare bacterial infections occurring after fat grafting procedures and emphasizes the importance of identifying the causative agent through advanced techniques such as DNA sequencing. Further research is needed to improve our understanding of the risks associated with cryopreservation of fat and to identify ways to prevent these types of infections in the future.

## Introduction

Breast augmentation through autologous fat-only grafting is a common procedure in the fields of plastic and aesthetic surgery. However, postoperative infections can occur and may lead to unsatisfactory results, including implant loss, breast deformation, and in rare cases, systemic infections (Groen et al., 2016; Ørholt et al., 2020). *Corynebacterium bovis*, a bacterium typically associated with bovine mastitis (Ajitkumar et al., 2012), has only been reported to cause rare infections in humans, with only 14 documented cases to date. These cases encompass a range of conditions, such as line-related sepsis, ventriculi jugular shunt nephritis, meningitis, leg ulcer, chronic otitis media, epidural abscess, keratitis, chronic conjunctivitis, shoulder prosthetic joint infection, and infectious endocarditis (Bolton et al., 1975; Vale and Scott, 1977; Dalal et al., 2008; Ajitkumar et al., 2012; Chow et al., 2015; Elsheikh et al., 2021).

Here, we present that a case of a patient who developed a C. bovis infection after autologous fat-only breast augmentation and describes the treatment process, as well as a discussion of possible causes of the infection. The infection was noted following asecondary fat grafting with cryopreserved fat from the initial operation, highlighting the potential risk of cryopreservation in the transmission of bacteria. To our knowledge, this marks the first reported case of C. bovis infection in the context of human fat breast augmentation.

In this paper, we delineate the treatment process for this uncommon infection and delve into potential sources of the infection, encompassing aspects like cryopreservation and the utilization of contaminated instruments during the surgical procedure. Furthermore, we propose the potential use of DNA sequencing to identify the causative organism in culture-negative infections. Our findings highlight the importance of considering rare bacterial species as potential pathogens in postoperative infections and suggest the need for further investigation into the safety of cryopreservation in fat grafting procedures.

## Case report

In May 2022, a 46-year-old female patient underwent thigh liposuction and subsequently fat breast augmentation. The liposuction procedure utilized a 3-mm multiport cannula, which incorporated several 1 mm sharp side holes and operated at a suction pressure of -0.75 atm. The duration of the procedure was approximately one hour, and the extracted fat was subsequently subjected to centrifugation at 1200 g for 3 minutes to produce Coleman fat. A total volume of 800ml of Coleman fat was prepared, with 300ml of Coleman fat being administered to each breast for augmentation purposes. The remaining fat was cryopreserved at -18°C without the inclusion of a cryoprotectant solution. The patient did not exhibit any discernible symptoms of fever or infection following the initial liposuction and lipofilling operations.

One month after post-breast augmentation surgery, the patient underwent a second procedure using residual fat due to dissatisfaction with the initial outcome. The total filling volume was 175ml, with 75ml in the left breast and 100ml in the right breast.

**Figure 1:**
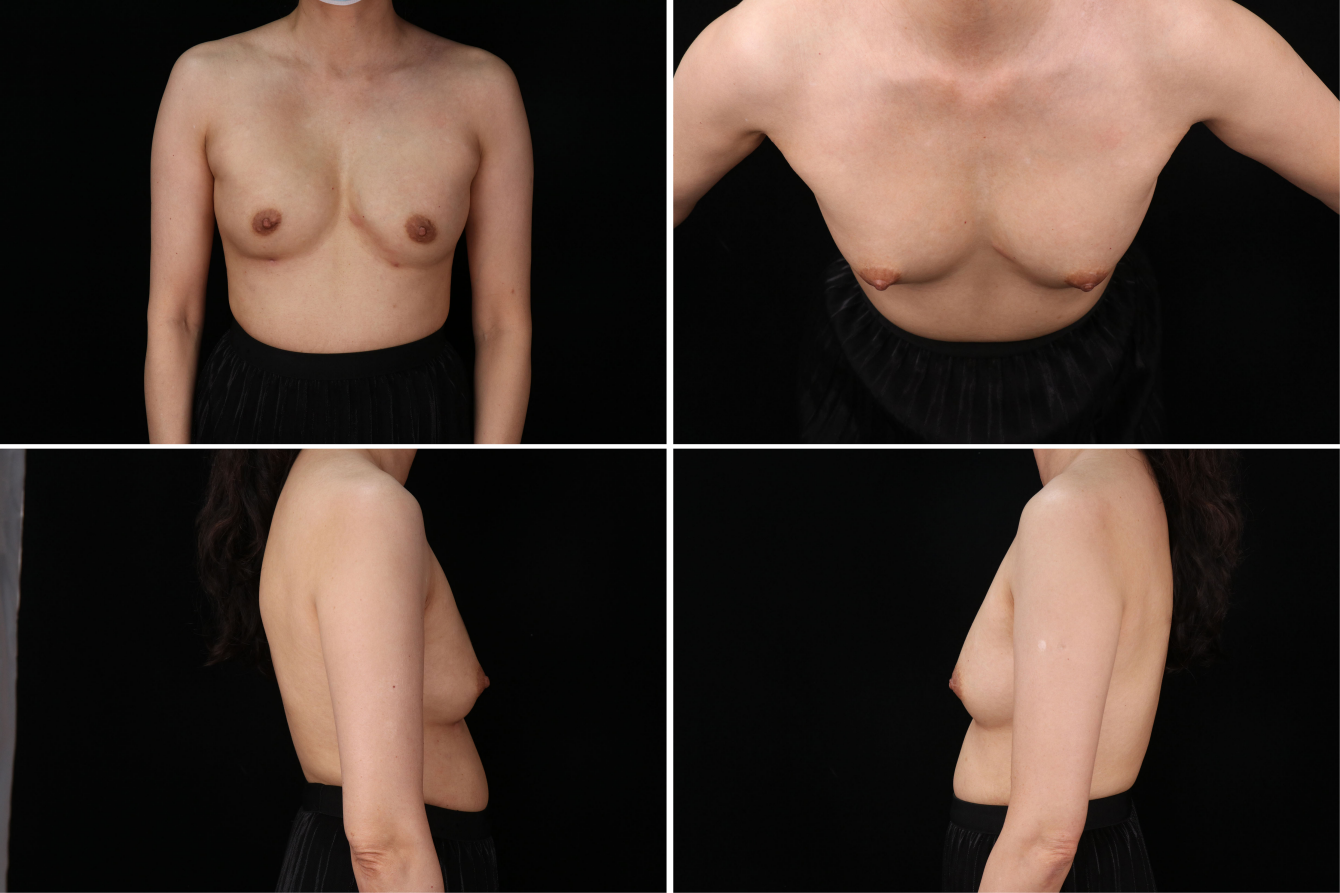
The preoperative photo of the breast, exhibited the existence of edema and indications of localized inflammation at the injection port.

**Figure 2:**
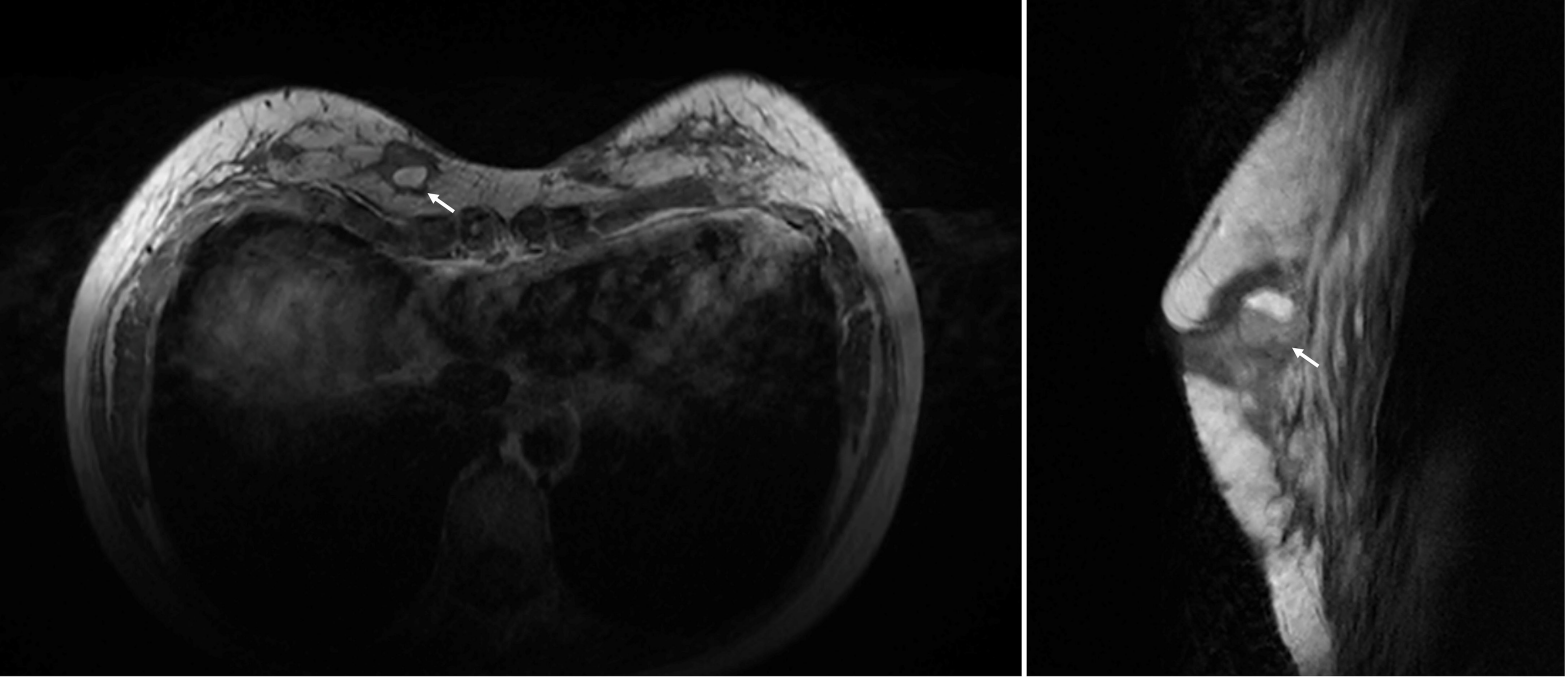
The preoperative photos of MRI images, revealed the presence of a circular abnormality measuring 2.0 x 2.0 x 1.4 cm in the right breast (white arrow).

Following the second procedure, the patient experienced localized redness and swelling at both filling ports, along with breast pain and swelling. Despite treatment with prednisone and antibiotics, the symptoms persisted and worsened over time, leading to referral to our hospital. Despite the absence of indications of systemic inflammation, notable symptoms of localized inflammation were observed, including swelling and pain in both breasts, along with local erythema and the discharge of purulent exudate from the injection port (**Figure 1**). Breast ultrasound detected the presence of multiple areas with no echo in both mammary glands, with the largest measuring 2.2 x 1.9 cm. Further MRI results revealed the existence of numerous adipose nodules at varying levels. Additionally, circular anomalous signal shadows, measuring approximately 2.0 x 2.0 x 1.4 cm, were visible in the lower quadrant of the right breast, indicative of infection and abscess formation (**Figure 2**).

To determine the causative agents, we employed high-throughput DNA sequencing technology. High-throughput DNA sequencing is a non-culture-based technique that enables direct nucleic acid sequencing of clinical samples, enabling the identification, tracing, detection, and typing of infectious pathogens through database comparison. Sample was extracted from the purulent exudate of the injection port and sent for sequencing. The sequencing analysis yielded a total of twelve bacterial sequences, while no evidence of fungi, viruses, parasites, or other pathogens was detected. Upon comparison with the database, all the bacterial sequences were confirmed as C. bovis. Due to the absence of residual fat, pathogenic testing was not possible.

To address the source of infection, we investigated the sterilization process and storage container that the residual fat was stored in. The sterilization process was performed in accordance with standard clinical procedures, and the storage container was a sterile polypropylene container. However, the fat was cryopreserved at -18°C without the inclusion of a cryoprotectant solution. Cryopreservation has been shown to cause damage to cell membranes and could result in bacterial contamination due to the formation of ice crystals, which could facilitate bacterial entry into the cells.

**Figure 3:**
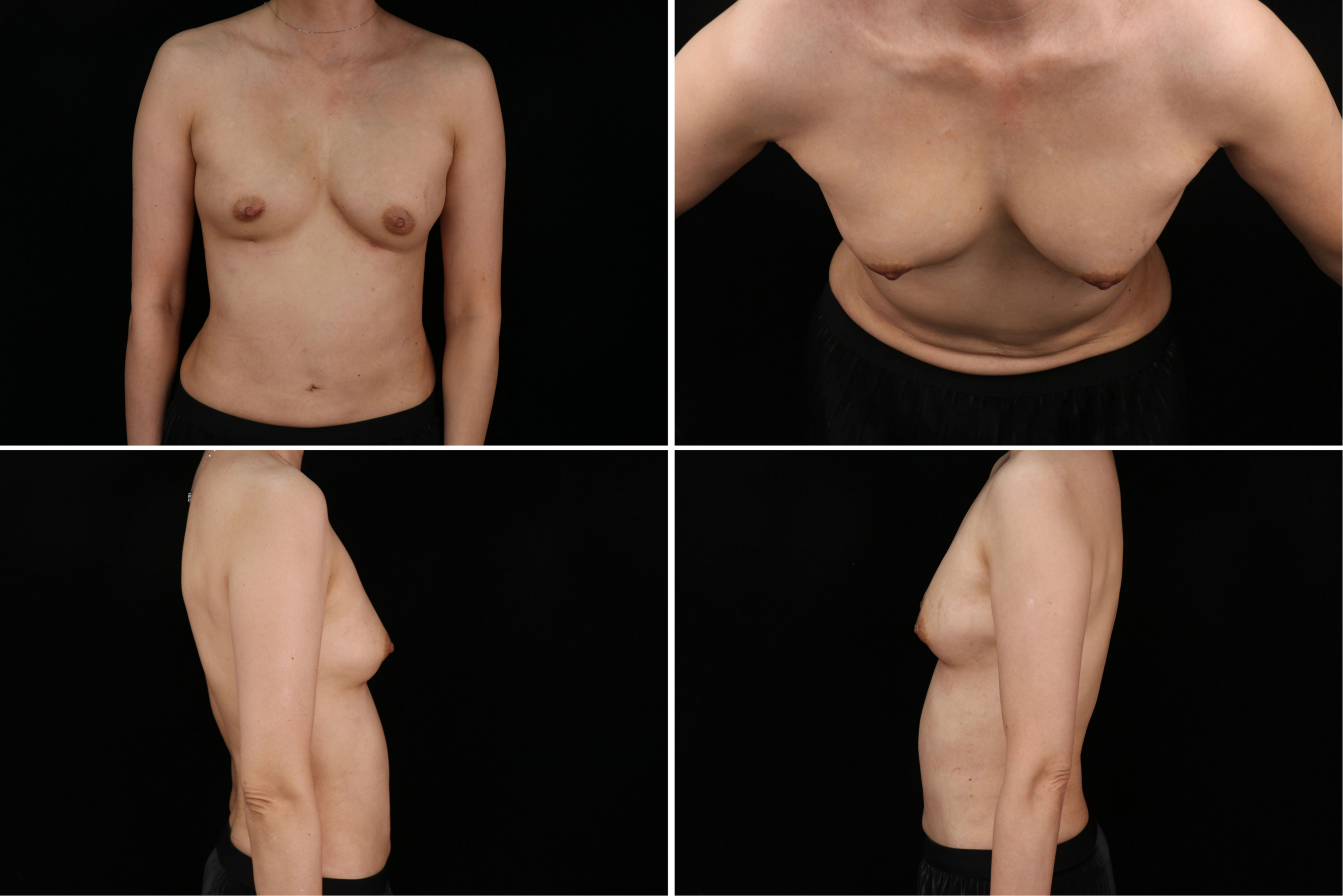
The photo of final clinical outcome, signs of local redness and swelling resolved.

**Figure 4:**
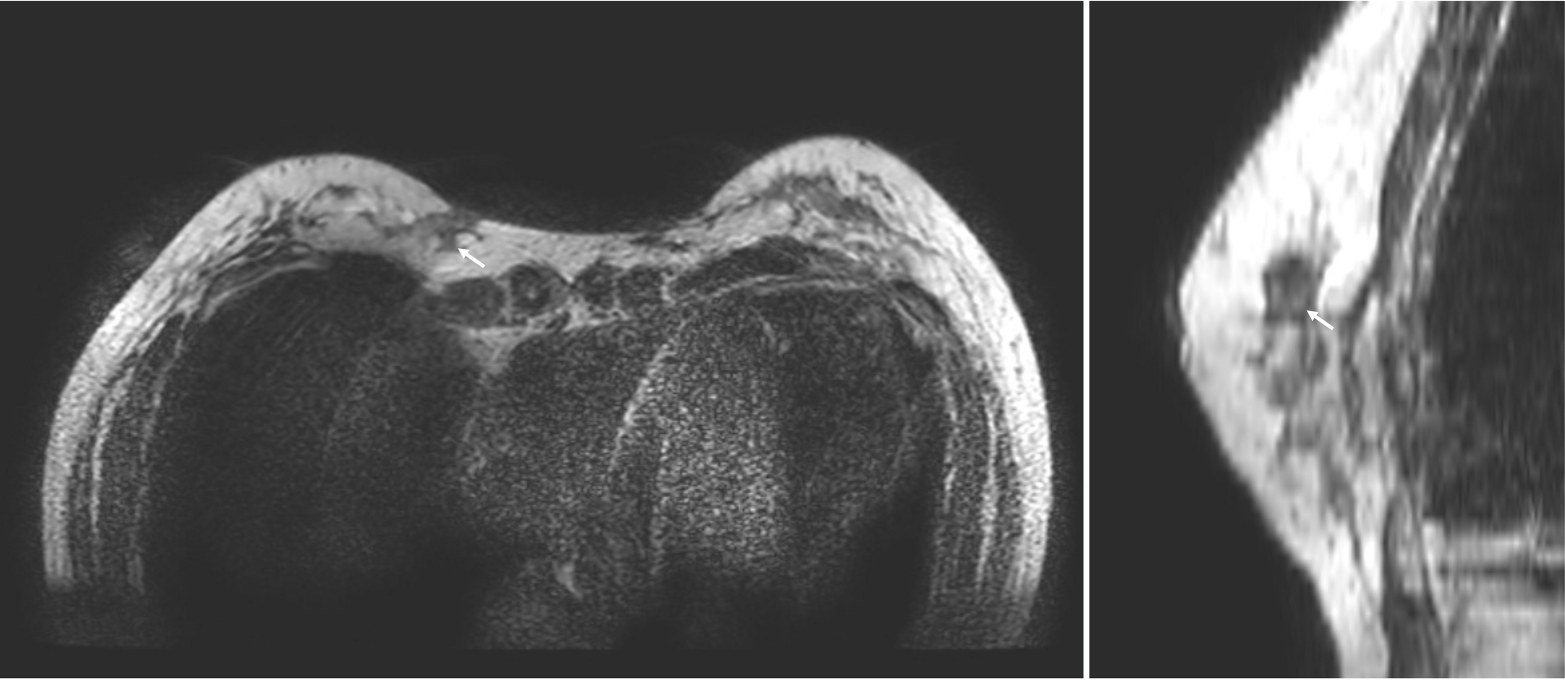
The photos of MRI images at 3 months after surgery, revealed the lesion is significantly smaller than before (white arrow).

In line with previous literature (Elsheikh et al., 2021), after undergoing debridement surgery, the patient was administered intravenous imipenem for a duration of one week in order to alleviate the breast infection. The turnaround time for the DNA sequencing test was five days. During the test, the patient was administered ceftriaxone and cefuroxime. Upon confirmation of the C. bovis infection, the patient was switched to imipenem. After the eight-week follow-up, no discernible clinical or laboratory indications of infection were detected, and the previously present local redness and swelling in the left breast had resolved (**Figure 3**). The MRI findings at 3 months after the operation indicated a notable decrease in the size of the infection site as compared to the earlier one (**Figure 4**).

## Discussion

Autologous fat grafting for breast augmentation is a common procedure in cosmetic surgery, but it can lead to various complications such as pain, hematoma formation, oil cyst development. Another challenge is that fat grafting has a high absorption rate and unstable volume retention rate. This can lead to unsatisfactory results, necessitating additional injections. To avoid multiple procedures, adipose tissue can be cryopreserved for future injections. However, there is no universally accepted technique for cryopreserving fat, although evidence suggests that an effective and appropriate cryopreservation method can maintain cellular activity and function (Gal and Pu, 2020).

Unfortunately, the patient in this case developed a C. bovis infection after the second injection of cryopreserved fat, which was suspected to be the source of infection. The infection’s likely cause was the use of cryopreserved fat harvested during the initial procedure and stored for four weeks before reinjection. The cryopreservation process involved slowly cooling the adipose tissue to -30°C before storing it in a refrigerator at the same temperature without any protective agent added to the fat. This creates an environment that may promote bacterial growth, as cryopreservation typically involves slowing down metabolic processes but does not completely eliminate the presence of microorganisms. Cryoprotective agents can be added to protect cells from the damaging effects of ice crystal formation during freezing. However, the subsequent removal of the protective agent can be challenging, requiring repeated cleaning, and increasing the risk of contamination and infection. To mitigate this risk, it’s crucial to implement proper protocols for cryopreservation, ensuring that the storage temperature and conditions are not conducive to microbial proliferation. Moreover, the use of sterile, well-sealed containers can reduce the risk of contamination during storage.

Moreover, there is also a possibility of infection during the supplemental injection process. Anytime a needle is inserted into the skin, there is a risk of introducing bacteria or other harmful pathogens into the body. Proper sterilization and disinfection of all equipment and the surgical field are vital to minimize this risk. The surgical team should adhere to stringent aseptic techniques and ensure that all equipment, including needles, syringes, and other instruments, are sterile. Although not analyzed in this particular case, the storage container and the sterilization process for residual fat should not be overlooked. The choice of storage containers can impact the integrity of the adipose tissue and the risk of contamination. Sterilization methods for equipment used during fat processing and storage should be thoroughly validated to ensure their effectiveness in eliminating microbial contaminants. Evaluating the container materials and sterilization procedures as potential sources of infection should be a part of comprehensive quality control measures in fat grafting procedures.

Regarding the DNA testing, the authors used fluid from the pocket for analysis. The absence of residual fat in the patient prevented pathogenic testing. The authors utilized high-throughput sequencing technology, which presents a unique advantage as it enables direct nucleic acid detection in clinical samples and subsequent comparison with an established database. For instance, Achermann et al. (2009) identified C. bovis as the pathogen by sequencing the 16S rRNA gene and comparing it with the gene database. The turnaround time for this DNA testing was five days. The turnaround time for the DNA sequencing test was five days. During the test, the patient was administered ceftriaxone and cefuroxime. Upon confirmation of the C. bovis infection, the patient was switched to imipenem.

C. bovis is a Gram-positive, facultatively anaerobic bacterium that is known to cause infrequent infections in humans. The etiology of C. bovis infection is often associated with traumatic injury or the introduction of exogenous medical devices. For example, Vale et al (Vale and Scott, 1977). found that out of a case series of six patients with C. bovis infections, two were associated with traumatic injury and two with the implantation of heart valves. In addition, Dalal et al. (2008) reported on an 84-year-old female who acquired a C. bovis infection through a peripherally inserted central catheter (PICC) following a cerebrovascular accident. Contact with contaminated cows or their products has also been identified as a possible source of C. bovis infection. For instance, Elsheikh et al. (2021) documented a case of persistent bacterial keratitis, where the patient had recent cattle contact, suggesting that hand-to-eye contact with infected cattle was the likely mode of transmission. Similarly, Achermann et al. (2009) described a case of shoulder prosthetic joint infection, where the author postulated that the infection may have been due to contaminated milk or the patient’s own skin flora.

This study reports the initial occurrence of C. bovis infection in a fat breast augmentation case. The etiology of the infection remains undetermined. The patient denied any exposure to livestock or contaminated cattle products and had no history of breast trauma. The preoperative evaluation revealed no signs of HIV infection or other forms of immunosuppression. Overall, our findings highlight the importance of considering unusual pathogens and performing comprehensive diagnostic workups when facing culture-negative infections. While traditional culture techniques remain the standard of care for identifying bacterial pathogens, the use of high-throughput sequencing technology can provide valuable information in cases of suspected unusual infections, enabling more targeted antibiotic treatment and potentially preventing future infections. High-throughput DNA sequencing, despite its numerous benefits in microbiology and infectious disease diagnosis, comes with several notable limitations. Firstly, its cost remains relatively high compared to traditional culture methods, limiting its adoption in resource-limited settings. The technique is also complex and requires specialized expertise in molecular biology and bioinformatics, which may hinder its widespread use. Turnaround time can be an issue, as it can still take hours to days to obtain results. Furthermore, the technology’s sensitivity can lead to false positives, and data interpretation is challenging due to the vast amount of information generated. Sample quality and preparation are crucial, and not all healthcare facilities have access to this technology. Ethical and legal concerns, particularly regarding patient privacy and data security, can also arise. Despite these limitations, ongoing advancements may address some of these issues, and high-throughput sequencing continues to hold promise for the future of infectious disease diagnosis.

## Conclusion

In conclusion, autologous fat grafting remains a popular cosmetic procedure despite the associated risks of complications. Our case report highlights the potential risks of using cryopreserved fat-derived products, which may increase the risk of infection and adipocyte necrosis. Furthermore, it underscores the importance of thorough preoperative evaluation and postoperative monitoring for early identification and treatment of complications. Finally, the use of high-throughput sequencing technology can be a valuable tool in diagnosing culture-negative infections and guiding antibiotic treatment. Further studies are needed to investigate the safety and efficacy of cryopreservation techniques for autologous fat grafting and to identify additional measures to prevent infections in cosmetic procedures.

## Data availability statement

The original contributions presented in the study are included in the article/supplementary material. Further inquiries can be directed to the corresponding author.

## Ethics statement

The studies involving humans were approved by Ethics Committee, Nanfang Hospital, Southern Medical University. The studies were conducted in accordance with the local legislation and institutional requirements. The participants provided their written informed consent to participate in this study. Written informed consent was obtained from the individual(s) for the publication of any potentially identifiable images or data included in this article. Written informed consent was obtained from the participant/patient(s) for the publication of this case report.

## Author contributions

XY: Writing – original draft. YY: Writing – original draft. JG: Writing – review & editing. YL: Writing – review & editing.

## References

[B1] Achermann Y. Trampuz A. Moro F. Wüst J. Vogt M. (2009). *Corynebacterium bovis* shoulder prosthetic joint infection: the first reported case. Diagn. Microbiol. Infect. Dis. 64 (2), 213–215. doi: 10.1016/j.diagmicrobio.2009.02.003 19304438

[B2] Ajitkumar P. Barkema H. W. De Buck J. (2012). Rapid identification of bovine mastitis pathogens by high-resolution melt analysis of 16S rDNA sequences. Vet. Microbiol. 155 (2-4), 332–340. doi: 10.1016/j.vetmic.2011.08.033 21944716

[B3] Bolton W. K. Sande M. A. Normansell D. E. Sturgill B. C. Westervelt F. B. Jr. (1975). Ventriculojugular shunt nephritis with Corynebacterium bovis. Successful Ther. antibiotics. Am. J. Med. 59 (3), 417–423. doi: 10.1016/0002-9343(75)90401-5 1163549

[B4] Chow S. K. Bui U. Clarridge J. E. (2015). *Corynebacterium bovis* eye infections, washington, USA, 2013. Emerg. Infect. Dis. 21 (9), 1687–1689. doi: 10.3201/eid2109.150520 26291771 PMC4550150

[B5] Dalal A. Urban C. Ahluwalia M. Rubin D. (2008). *Corynebacterium bovis* line related septicemia: a case report and review of the literature. Scand. J. Infect. Dis. 40 (6-7), 575–577. doi: 10.1080/00365540701772448 18584551

[B6] Elsheikh M. Elsayed A. Bennett N. Connor M. (2021). *Corynebacterium bovis*: A rare case of persistent bacterial keratitis and corneal perforation. Cureus 13 (8), e16913. doi: 10.7759/cureus.16913 34513486 PMC8412843

[B7] Gal S. Pu L. L. Q. (2020). An update on cryopreservation of adipose tissue. Plast. Reconstr Surg. 145 (4), 1089–1097. doi: 10.1097/PRS.0000000000006699 32221240

[B8] Groen J. W. Negenborn V. L. Twisk J. W. Ket J. C. Mullender M. G. Smit J. M. (2016). Autologous fat grafting in cosmetic breast augmentation: A systematic review on radiological safety, complications, volume retention, and patient/surgeon satisfaction. Aesthet Surg. J. 36 (9), 993–1007. doi: 10.1093/asj/sjw105 27329661

[B9] Ørholt M. Larsen A. Hemmingsen M. N. Mirian C. Zocchi M. L. Vester-Glowinski P. V.. (2020). Complications after breast augmentation with fat grafting: A systematic review. Plast. Reconstr. Surg. 145 (3), 530e–537e. doi: 10.1097/PRS.0000000000006569 32097306

[B10] Vale J. A. Scott G. W. (1977). *Corynebacterium bovis* as a cause of human disease. Lancet 2 (8040), 682–684. doi: 10.1016/s0140-6736(77)90495-0 71496

